# Combination of C-reactive protein, procalcitonin and sepsis-related organ failure score for the diagnosis of sepsis in critical patients

**DOI:** 10.1186/s13613-016-0153-5

**Published:** 2016-06-10

**Authors:** Yi Yang, Jianfeng Xie, Fengmei Guo, Federico Longhini, Zhiwei Gao, Yingzi Huang, Haibo Qiu

**Affiliations:** Department of Critical Care Medicine, Nanjing Zhong-Da Hospital, School of Medicine, Southeast University, Nanjing, 210009 Jiangsu China; Department of Translational Medicine, Eastern Piedmont University “A. Avogadro”, Novara, Italy

**Keywords:** C-reactive protein, Procalcitonin, Interleukin-6, Sepsis, Bioscore

## Abstract

**Objective:**

To measure the ability of a new bioscore to diagnose sepsis in a general critical care population.

**Methods:**

The study was done at an intensive care unit (ICU) from April to December 2012. Demographic and clinical patient information were recorded on admission to the ICU with blood samples taken for C-reactive protein (CRP), procalcitonin (PCT), interleukin-6, white blood cell count, as well as body temperature, age and the sepsis-related organ failure (SOFA) score. These parameters were used to create a scoring system. The scoring system then underwent analysis by univariate analysis and multivariate logistic regression analysis to identify which of these clinical parameters were statistically different in septic versus non-septic patients. The bioscore was then tested in a receiver operator characteristic curve to determine statistical significance of the scoring systems ability to predict sepsis. Finally, a bioscore cutoff value was defined to provide a level for sepsis diagnosis.

**Results:**

Three hundred patients were enrolled, of which 107 patients were septic and 193 patients were non-septic. Univariate logistic regression showed that age, gender, CRP, PCT and SOFA were risk factors for occurrence of sepsis. Multivariate analysis revealed CRP (AUC 0.729, 95 % CI 0.671–0.787, *P* < 0.001), PCT (AUC 0.711, 95 % CI 0.652–0.770, *P* < 0.001) and SOFA (AUC 0.670, 95 % CI 0.607–0.733, *P* < 0.001) to be statistically significant. The combination of these values in the bioscore had an AUC of 0.790 (95 % CI 0.739–0.834, *P* < 0.001). A bioscore of ≥2.65 was considered to be statistically significant in making a positive diagnosis of sepsis.

**Conclusions:**

This bioscore using CRP, PCT and SOFA score may potentially be used in the future to help identify septic patients earlier, improving their access to timely treatment modalities.

**Electronic supplementary material:**

The online version of this article (doi:10.1186/s13613-016-0153-5) contains supplementary material, which is available to authorized users.

## Background

Sepsis and septic shock are pathological conditions impacting a large proportion of patients admitted to the ICU, and these patients unfortunately have poor outcomes [1]. Sepsis results from a dysregulated host response to infection leading to uncontrolled inflammation and organ dysfunction and potentially a hypotensive state known as septic shock [2]. This clinical scenario falls under the multiple organ dysfunction syndrome [3].

Early diagnosis and direct appropriate therapy within the first hours of hospital admission has been shown to have beneficial effects with respect to patient outcome [4]. Furthermore, diagnostic and treatment delay prolongs hospital length of stay and increases healthcare costs [5, 6]. As a result, the availability of an efficient biomarker/evaluation system would be crucial to help diagnose sepsis quickly. To be useful, a biomarker should be characterized by its “capacity to provide timely information beyond that which is readily available from routine physiologic data and clinical examination” [7].

Several bloodstream biomarkers in sepsis have been previously investigated [8], including PCT and CRP [9]. Sepsis-induced cytokines promote the production of PCT, which is typically secreted by C cells of the thyroid in response to hypercalcemia. Similarly, CRP is an acute-phase reactant, synthesized by the liver, mainly in response to IL-6. IL-6 is a cytokine that generates an initial response to injury or infection; its levels rise significantly during early sepsis, and for this reason, it has been used for sepsis diagnosis and patient outcome prediction [10, 11]. Despite their common use, all these biomarkers suffer individual applicability limitations, including lack of sepsis specificity [12]. Another approach has been to consider a combination of markers and clinical parameters, known as a bioscore. Recently, Gibot et al. [13] demonstrated high diagnostic performance by a bioscore that combined the intensity of CD64 expression on polymorphonuclear cells (PMN CD64 index) together with PCT and the soluble triggering receptor expressed on myeloid cells-1 (sTREM-1) serum levels. However, while PCT measurement is widely and easily performed, sTREM-1 determination (by an ELISA assay) and CD64 assessment (by flow cytometery) is not routinely available in all hospitals, giving some limitations to this approach.

We hypothesized that by combining the common clinical biomarkers CRP, PCT and IL-6 with other clinical information, such as WBC, body temperature, age and gender, and the SOFA score in an extended bioscore (e-BS), we might be able to increase sensitivity and specificity of the combination of these tools for sepsis diagnosis. The aim of this investigation was to evaluate the value of these individual markers and to investigate whether an e-BS might have potential to improve early sepsis diagnosis.

## Methods

### Study design

This prospective study was performed in the ICU of a Nanjing Zhong-Da Hospital from April to December 2012. The protocol was approved by the local Institutional Ethics Committee (Approval Number: 2012ZD11KY08.0) in accordance with the ethical standards of the Declaration of Helsinki. Patients admitted into the ICU were enrolled in this study following informed consent from the patient or their guardian.

### Patients

Inclusion criteria were: (1) ICU admission; (2) signed informed consent from the patient or their guardian; (3) ≥18 years of age. Patients were excluded from enrollment if they had been hospitalized or received antibiotics in the preceding 2 weeks, or if it was an ICU re-admission.

All eligible patients were treated by the attending physician according to normal clinical practice. If an infection was suspected or documented, samples of organic fluids were collected for microbiologic cultural tests and antimicrobial therapy was prescribed according to the ICU practice and guidelines [14] without any intervention by the researchers.

Sepsis was clinically defined as a diagnosed infection and at least two of four systemic inflammatory response syndrome (SIRS) criteria [15] which include: (a) body temperature >38 or <36 °C, (b) heart rate >90 beats/min, (c) respiratory rate >20 breaths/min or an arterial partial pressure of carbon dioxide < 4.3 kPa (32 mmHg), (d) white blood cell count >12,000 or <4000/mm^3^, or the presence of >10 % immature neutrophils. The infection was defined on the basis of infection sites, clinical features, clinical microbiology and imaging tests. Two intensive care physicians were asked to retrospectively and independently analyze the clinical documentation for each patient, signs, symptoms and recent medical history to make the diagnosis of sepsis.

### Data collection

The acquired dataset included the patients’ demographic information, reason for admission, department of origin, infection site, blood culture results and laboratory characteristics. The patients were subsequently followed for 28 days for mortality.

Acute physiology and chronic health evaluation II (APACHE II) and sepsis-related organ failure assessment (SOFA) scores were calculated using data from the first 24 h after admission. Clinically significant WBC count was defined as WBC <4000 cells/µL (leukopenia) or >12,000 cells/µL (leukocytosis). Clinically significant body temperature was defined as <36 °C (hypothermia) or >38 °C (hyperthermia) [14, 15]. We also recorded the ICU and hospital length of stay.

### Measurement of CRP, PCT and IL-6

Within 12 h after ICU admission, 5–10 mL of blood was sampled for CRP, PCT and IL-6 measurements. CRP concentrations were measured in a serum sample, using a turbidimetric immunoassay test (BNII, Siemens Healthcare Diagnostic, Germany). PCT concentrations were assessed in a serum sample, using an immunoassay with a sandwich technique and a chemiluminescent detection system, while IL-6 concentrations using a turbidimetric immunoassay test (Roche Elecsys^®^ and MODULAR^®^ E170, Switzerland). CRP, PCT and IL-6 were transformed into categorical score values (Additional file 1: Table S1).

### Statistics and the scoring system

Normality distribution for quantitative variables was tested by the Kolmogorov–Smirnov test (*P* > 0.10). For categorical variables, Chi-square test, Fisher’s exact test or McNemar test were applied as appropriate. Comparison of continuous variables between the two groups was conducted with Student’s *t* test or Mann–Whitney *U* test depending on Gaussian distribution. To compare data from three or more patient groups, we applied the one-way analysis of variance (ANOVA), and, when indicated, the Student–Newmann–Keuls method was used as a post hoc test. Sensitivity and specificity were computed for all biomarker assays.

Logistic regression was used according to the presence or absence of sepsis and death as dependent variables to calculate the corresponding regression coefficients (using round numbers). The bioscore system was analyzed for the area under the curve, and the subsequent ROC curves were used to evaluate the prognostic value. The Youden index was applied to set the cutoffs and compared between the combined evaluation method and single evaluation methods.

Normally distributed data are expressed as mean ± SD, while non-normal distributed as median [25th–75th IQR]. All statistical analyses were performed using SPSS 19.0 software (IBM SPSS, USA); two-tailed *P* values <0.05 were considered statistically significant.

## Results

### Baseline data

Three hundred consecutive patients were included. The entire population characteristics including infection and organism findings of septic populations are shown in Table 1 and Additional file 1: Table S2. Specifically, we collected data on age, gender, core body temperature, WBC, PCT, CRP, IL-6 and calculated a SOFA score. Of note, 107 (35.7 %) patients (septic population) were septic, which included the subgroups of sepsis (*n* = 28, 9.3 %), severe sepsis (*n* = 41, 13.7 %) or septic shock (*n* = 38, 12.7 %) patients. The other 193 patients were not septic.

**Table 1:** Whole population characteristics and difference between septic and non-septic patients

Characteristics	Total 300	Septic patients 107 (36 %)	Non-septic patients 193 (64 %)	*P* value
Age (years)	64 ± 18	69 ± 15	61 ± 19	<0.001
Male sex, *n* (%)	173 (58)	74 (69)	99 (51)	<0.001
Body temperature, *n* (%)				0.03
<36 or >38 °C	97 (32.3 %)	43 (40.2)	54 (28.0)	
36–38 °C	203 (67.7 %)	64 (59.8)	139 (72.0)	
APACHE II score	17 ± 8	19 ± 7	16 ± 8	0.002
SOFA score	6.8 ± 3.5	8.2 ± 3.5	6.1 ± 3.2	<0.001
Admission department—no. (%)				
Emergency room	86 (28.7)	39 (36.4)	47 (24.4)	0.026
Surgical wards	164 (54.7)	32 (29.9)	132 (68.4)	<0.001
Without surgical procedure	5 (1.7)	3 (2.8)	2 (1.0)	0.252
With surgical procedure	159 (53)	29 (27.1)	130 (67.3)	<0.001
Medical wards	50 (16.7)	36 (33.6)	14 (7.2)	<0.001
Pathology of admission—no. (%)				
Acute pancreatitis	2 (0.7)	1 (0.9)	1 (0.5)	1.0
Intracerebral hemorrhage	32 (10.7)	8 (7.5)	24 (12.4)	0.183
Cardiac arrest	8 (2.7)	2 (1.9)	6 (3.1)	0.519
Trauma	17 (5.7)	3 (2.8)	15 (7.8)	<0.001
Acute myocardial infarction	10 (33.3)	4 (3.7)	6 (3.1)	0.777
High-risk surgery	130 (43.3)	0	130 (67.4)	
Cardiac surgery	51 (17.0)	0	51 (26.4)	
Non-cardiac surgery	79 (26.3)	0	79 (40.9)	
Sever sepsis	79 (26.3)	79 (73.8)	0	
Other	22 (7.3)	10 (9.3)	12 (6.1)	0.296
CRP	37.25 (8.79–87.13)	63.8 (32.3–114)	21.8 (5.20–60.55)	<0.001
PCT	0.66 (0.16–3.13)	1.79 (0.57–8.95)	0.38 (0.11–1.54)	<0.001
IL-6	125.2 (52.5–463.3)	124.8 (43.9–418.3)	125.6 (54.5–479.9)	0.97
Length of ICU stay—no. of days	5 [2–10]	8 [4–14]	3 [1–8]	<0.001
Length of hospital stay—no. of days	19 [10–28]	23 [13–32]	18 [9–26]	0.005
28-day mortality—no. (%)	75 (25)	32 (30)	43 (22)	0.144

### Diagnostic value of the scoring system

**Fig. 1:**
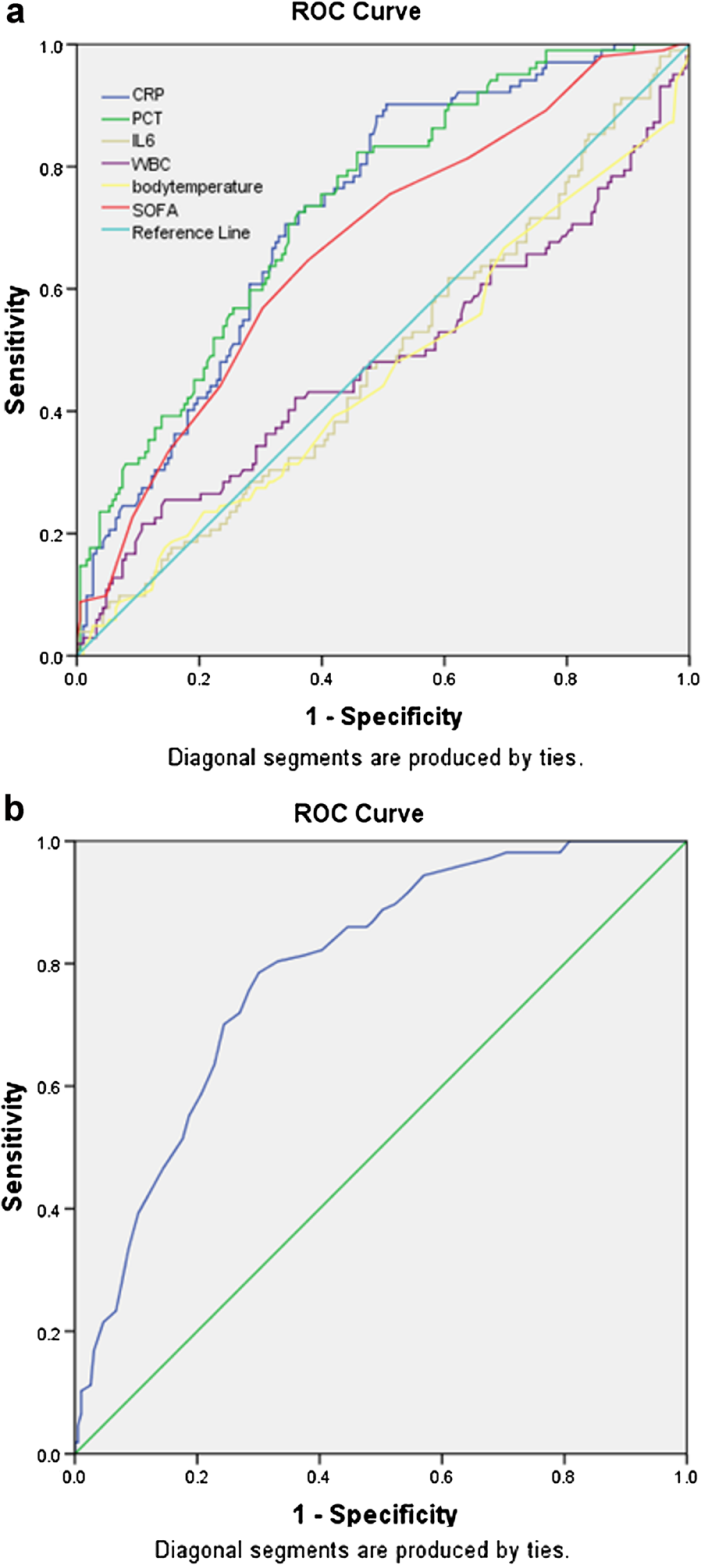
Diagnostic values for sepsis of clinical variables estimated by receiver operating curve (ROC) analysis. **a** Area under the curve (AUC) for a null hypothesis was 0.5. AUC for C-reactive protein (CRP) was 0.729 (95 % CI 0.675–0.779, *P* < 0.001), procalcitonin (PCT) 0.737 (95 % CI 0.683–0.786, *P* < 0.001), interleukin-6 (IL-6) 0.511 (95 % CI 0.452–0.570, *P* = 0.760), white blood cells (WBC) 0.472 (95 % CI 0.400–0.545, *P* = 0.426), body temperature 0.468 (95 % CI 0.398–0.539, *P* = 0.362) and sepsis-related organ failure assessment (SOFA) 0.671 (95 % CI 0.615–0.714, *P* < 0.001). **b** For calculated score: Area under the curve (AUC) for a null hypothesis was 0.5. AUC for calculated score was 0.790 (95 % CI 0.739–0.834, *P* < 0.001)

Univariate analysis of the measured raw patient parameters using a ROC curve showed that CRP, PCT and SOFA were significant for the diagnosis of sepsis (Fig. 1a). The AUCs were 0.729, 0.737 and 0.671, respectively (Table 2). The analysis revealed that clinical laboratory values of 21.1, 0.4475 and 6, respectively, were cutoff points that helped make the diagnosis of sepsis. However, given that the AUC values were low, we felt using the raw laboratory values would not accurately be able to predict a sepsis diagnosis. Therefore, we used the measured parameters to define a categorical-based scoring system in an attempt to better delineate syndrome clusters.

**Table 2:** Diagnostic value of each parameter in sepsis

Variables	AUC	*P* value	Sensitivity	Specificity	+LR	−LR
All patients						
SOFA	0.671	<0.001	66.4	61.7	1.73	0.55
CRP	0.729	<0.001	90.7	49.7	1.80	0.19
PCT	0.737	<0.001	82.9	53.9	1.80	0.32
IL-6	0.511	0.760	28.2	78.7	1.32	0.91
Score	0.790	<0.001	78.5	70.0	2.61	0.31
Surgical patients						
SOFA	0.587	0.150	51.7	62.6	1.38	0.77
CRP	0.697	<0.001	89.7	45.0	1.63	0.23
PCT	0.698	<0.001	65.5	71.0	2.26	0.49
IL-6	0.522	0.713	75.9	36.4	1.19	0.66
Score	0.745	<0.001	82.8	63.4	2.26	0.27
Non-surgical patients						
SOFA	0.684	<0.001	71.8	59.7	1.78	0.47
CRP	0.762	<0.001	91.0	59.7	2.26	0.15
PCT	0.810	<0.001	75.0	77.4	3.32	0.32
IL-6	0.503	0.953	68.1	43.3	1.20	0.74
Score	0.836	<0.001	78.2	77.4	3.46	0.28
Newly hospitalized patients						
SOFA	0.781	<0.001	84.6	66.0	2.49	0.23
CRP	0.805	<0.001	89.7	66.0	2.64	0.16
PCT	0.868	<0.001	84.6	80.9	4.42	0.19
IL-6	0.601	0.101	82.1	44.7	1.48	0.40
Score	0.896	<0.001	84.6	83.0	4.97	0.19

In order to define the scoring system (bioscore), each measured clinical variable (using a rounded number) was converted into categorical values as a final weighted value, as shown in Additional file 1: Table S3. Subsequent univariate analysis of these ordinal values indicated that age, gender, PCT, CRP and SOFA were significantly correlated with occurrence of sepsis according to the score in Additional file 1: Table S1 (Table 3). However, we did not find WBC, body temperature and IL-6 had diagnostic value in recognizing sepsis. Then, multivariate analysis by logistic regression using the presence or absence of sepsis as a dependent variable and the parameters identified by univariate analysis found statistical significance of age, gender, CRP, PCT and SOFA (Table 3).

**Table 3:** Univariate and multivariate associations of PCT, CRP, SOFA score with sepsis

Marker	Univariate		Multivariate	
	OR (95 % CI)	*P*	OR (95 % CI)	*P*
Age^a^	3.171 (1.833, 5.485)	<0.001	2.662 (1.382, 5.127)	0.003
Gender^a^	0.470 (0.285, 0.773)	0.003	0.377 (0.203, 0.701)	0.002
WBC	0.871 (0.540, 1.405)	0.571	0.704 (0.378, 1.311)	0.268
PCT				
0	1.0 (Reference)		1.0 (Reference)	
1^a^	4.169 (2.158, 8.055)	<0.001	3.485 (1.561, 7.780)	0.008
2	4.345 (2.155, 8.761)	<0.001	3.550 (1.467, 8.593)	0.074
3^a^	8.369 (3.815, 18.359)	<0.001	6.501 (2.326, 18.173)	0.001
CRP				
0	1.0 (Reference)		1.0 (Reference)	
1	3.191 (1.453, 7.011)	0.004	2.491 (0.958, 6.477)	0.061
2^a^	7.700 (3.138, 18.894)	<0.001	6.795 (2.188, 21.096)	0.001
3^a^	8.800 (3.964, 19.537)	<0.001	6.593 (2.395, 18.125)	<0.001
SOFA	1.204 (1.117, 1.297)	<0.001	2.314 (1.260, 14.251)	0.007
IL-6				
0	1.0 (Reference)		1.0 (Reference)	
1	1.691 (0.586, 4.877)	0.331	1.380 (0.343, 5.559)	0.651
2	1.364 (0.468, 3.976)	0.569	0.639 (0.152, 2.678)	0.540
3	1.524 (0.526, 4.415)	0.438	0. 576 (0.146, 2.278)	0.432
4	1.227 (0.425, 3.542)	0.706	0. 422 (0.106, 1.681)	0.221

^a^Cox and Snell *R* ^2^ was 0.282, and Nagelkerke *R* ^2^ was 0.389

To further refine the scoring system, we eliminated age and gender as we felt these parameters were less specific to predicting when a patient was truly septic. Therefore, we analyzed the total bioscore of each patient (using only CRP, PCT and SOFA score) on a ROC. When the bioscore was plotted for analysis by ROC curve (Fig. 1b), the AUC was 0.790 (95 % CI 0.739–0.834, *P* < 0.001) with a cutoff value of 2.65 suggesting a diagnosis of sepsis above this value. The sensitivity and specificity of the scoring system were 78.5 and 70 %, respectively, with a positive likelihood ratio of 2.61 and negative likelihood ratio of 0.31, respectively (Table 2).

### Diagnostic value of the scoring system in subgroup populations

**Fig. 2:**
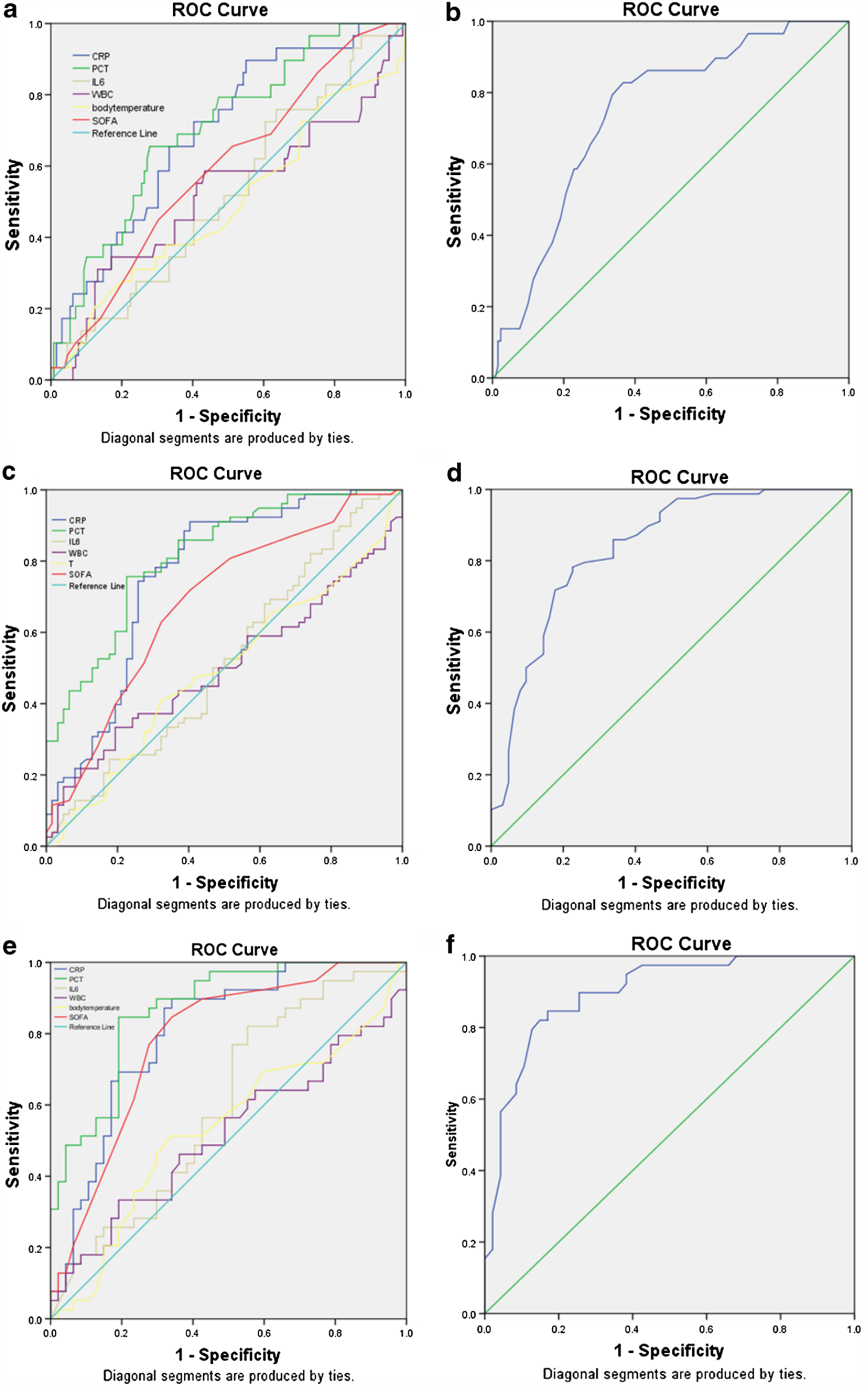
Diagnostic values for sepsis patients estimated by receiver operating curve (ROC) analysis. **a** For clinical variables in non-surgically treated patients: Area under the curve (AUC) for a null hypothesis was 0.5. AUC for C-reactive protein (CRP) was 0.697 (95 % CI 0.620–0.767, *P* < 0.001), procalcitonin (PCT) 0.698 (95 % CI 0.620–0.768, *P* < 0.001), interleukin-6 (IL-6) 0.522 (95 % CI 0.441–0.602, *P* = 0.713), white blood cells (WBC) 0.525 (95 % CI 0.445–0.604, *P* = 0.676), body temperature 0.511 (95 % CI 0.431–0.591, *P* = 0.851) and sepsis-related organ failure assessment (SOFA) 0.587 (95 % CI 0.507–0.664, *P* < 0.001). **b** For calculated score in non-surgically treated patients: Area under the curve (AUC) for a null hypothesis was 0.5. AUC for calculated score was 0.745 (95 % CI 0.670–0.810, *P* < 0.001). **c** For clinical variables in non-surgically treated patients: Area under the curve (AUC) for a null hypothesis was 0.5. AUC for C-reactive protein (CRP) was 0.762 (95 % CI 0.683–0.830, *P* < 0.001), procalcitonin (PCT) 0.810 (95 % CI 0.734–0.871, *P* < 0.001), interleukin-6 (IL-6) 0.503 (95 % CI 0.415–0.591, *P* = 0.953), white blood cells (WBC) 0.486 (95 % CI 0.390–0.582, *P* = 0.775), body temperature 0.478 (95 % CI 0.382–0.575, *P* = 0.659) and sepsis-related organ failure assessment (SOFA) 0.684 (95 % CI 0.600–0.769, *P* < 0.001). **d** For calculated score in non-surgically treated patients: Area under the curve (AUC) for a null hypothesis was 0.5. AUC for calculated score was 0.836 (95 % CI 0.764–0.893, *P* < 0.001). **e** For clinical variables in newly hospitalized sepsis patients: Area under the curve (AUC) for a null hypothesis was 0.5. AUC for C-reactive protein (CRP) was 0.805 (95 % CI 0.705–0.882, *P* < 0.001), procalcitonin (PCT) 0.868 (95 % CI 0.778, 0.931, *P* < 0.001), interleukin-6 (IL-6) 0.601 (95 % CI 0.490–0.705, *P* = 0.102), white blood cells (WBC) 0.482 (95 % CI 0.354–0.610, *P* = 0.774), body temperature 0.505 (95 % CI 0.378–0.632, *P* = 0.940) and sepsis-related organ failure assessment (SOFA) 0.781 (95 % CI 0.679–0.863, *P* < 0.001). **f** For calculated score in newly hospitalized sepsis patients: Area under the curve (AUC) for a null hypothesis was 0.5. AUC for calculated score was 0.896 (95 % CI 0.812–0.952, *P* < 0.001)

Surgical procedures impact physiologic parameters and, therefore, sought to evaluate the predictive ability of this bioscore in a subset of surgical patients. In a subgroup of patients that underwent surgical procedures (*n* = 160), univariate analysis determined significance for only CRP and PCT in sepsis diagnosis (Fig. 2a; Table 2). The AUC of CRP and PCT was 0.697 and 0.698, respectively. When the previously determined bioscore system was evaluated by ROC curve analysis in this patient subgroup, the AUC was 0.745 (95 % CI 0.670–0.810, *P* < 0.001) with a sensitivity and specificity of 82.8 and 63.4 %, respectively. The scoring system, which contained more contributing measurable data points, was higher than any of the individual parameters (Fig. 2b; Table 2).

In the non-surgically treated patients (*n* = 140), CRP, PCT and SOFA score had significant diagnostic value in diagnosing sepsis (Fig. 2c; Table 2). When the scoring system was evaluated by ROC curve analysis in this patient subgroup, the AUC was 0.836 (95 % CI 0.764–0.893, *P* < 0.001), which was better at identifying sepsis than any of the individual parameters (Fig. 2d; Table 2).

In the newly hospitalized patient subgroup (*n* = 86), univariate analysis revealed that CRP, PCT and SOFA were statistically significant in diagnosing sepsis (Fig. 2e; Table 2). Bioscores in this subgroup gave an AUC of 0.896 (95 % CI 0.812–0.952, *P* < 0.001) also showing a greater ability at identifying sepsis than any individual parameter (Fig. 2f; Table 2).

### Risk factors for the 28-day mortality

Univariate analysis detected age, body temperature, SOFA score and IL-6 demonstrating significance association with the 28-day mortality (OR 1.017, 95 % CI 1.001–1.033, *P* = 0.038; OR 1.214, 95 % CI 1.002–1.471, *P* = 0.048; OR 1.261, 95 % CI 1.160–1.371, *P* < 0.001; OR 1.010, 95 % CI 1.001–1.017, *P* = 0.045, respectively). Multivariate logistic regression analysis showed that only age and SOFA were independently associated with 28-day mortality (OR 1.022, 95 % CI 1.003–1.041, *P* = 0.024; OR 1.263, 95 % CI 1.148–1.389, *P* < 0.001) (Table 4). Cox and Snell *R*^2^ was 0.144, and Nagelkerke *R*^2^ was 0.215.

**Table 4:** Univariate and multivariate analysis of 28-day mortality

Marker	Univariate		Multivariate	
	OR (95 % CI)	*P*	OR (95 % CI)	*P*
Age^a^	1.017 (1.001, 1.033)	0.038	1.022 (1.003, 1.041)	0.024
Gender	0.670 (0.389, 1.156)	0.150	0.805 (0.437, 1.485)	0.488
PCT (ng/mL)	0.999 (0.996, 1.003)	0.671	0.997 (0.982, 1.013)	0.720
CRP (mg/L)	1.001 (0.997, 1.005)	0.606	0.999(0.994, 1.004)	0.718
SOFA^a^	1.261 (1.160, 1.371)	<0.001	1.263 (1.148, 1.389)	<0.001
IL-6	1.010 (1.001, 1.017)	0.045	1.000 (1.000, 1.001)	0.128
WBC	1.007 (0.997, 1.018)	0.179	1.005 (0.994, 1.004)	0.392
Body temperature	1.214 (1.002, 1.471)	0.048	1.100 (0.876, 1.381)	0.413

^a^Cox and Snell *R* ^2^ was 0.144, and Nagelkerke *R* ^2^ was 0.215

## Discussion

It is now generally agreed that a single clinical biomarker is not acceptable for accurately diagnosing and predicting prognosis during sepsis. The aim of this investigation was to evaluate the ability of an enhanced bioscore, where common clinical biomarkers are combined with other clinical laboratory information, to provide a more reliable diagnosis and prediction tool for sepsis patients. The study combined the most common clinical biomarkers for sepsis at ICU admission and clinical scoring methods to evaluate the possibility of increasing the accuracy of sepsis diagnosis in a general population of a Chinese ICU. The results of this study show that when threshold values of CRP, PCT and SOFA were taken into consideration by calculation of a bioscore value, this could be considered a statistically significant predictor for sepsis diagnosis. In these cases, the AUC values for the combined parameter score were more predictive than any one individual marker. Therefore, we consider these methods to have value in predicting sepsis.

CRP, PCT and IL-6 are used widely in attempting to clinically diagnose sepsis. In keeping with previous published data, our results confirm a low diagnostic value of CRP in a general ICU population [16]. However, when evaluating our entire study population, the accuracy of PCT was relatively low compared to previous studies and improved only slightly after selecting out surgical patients. In addition, we found that IL-6 suffered low diagnostic significance. There have also been contrasting results of IL-6 in diagnosing sepsis in the previous work, which may have been a result of different baseline patient characteristics [17–19].

Since CRP, PCT and IL-6 had been demonstrated to be elevated by various conditions without infection, i.e., trauma, surgery, burn, pancreatitis [20–22], we decided to analyze the subgroup populations [13]. We found a low diagnostic value of CRP and PCT in surgical patients and a high diagnostic value in non-surgical patients, especially in newly admitted patients. Our population was similar to the one described by Gibot et al. [13], and the diagnostic value of the previously mentioned biomarkers was comparable in their subgroup patients. However, we found the value of IL-6 in diagnosing sepsis did not change in the subgroup population, indicating IL-6 may not be a valuable biomarker for our cohort of patients.

Other factors widely assessed in the diagnosis of sepsis and determining prognosis are body temperature and WBC count. However, these factors have repeatedly being shown to have poor sensitivity and specificity for sepsis diagnosis [23, 24]. Similarly, we found a low diagnostic value of body temperature and WBC count in our analysis.

Single parameter measurement had a relatively low diagnostic value in sepsis. Therefore, we combined multiple clinical parameters to build a scoring system to improve diagnostic abilities. Recently, Gibot and colleagues reported that some relatively new biomarkers (PCT, sTREM-1 and PMN CD64 index) were useful for diagnosing sepsis and that the combination of them in a score had an impressive diagnostic accuracy [13]. However, as the authors stated, these measurements may not be routinely available in all hospitals, limiting the scoring systems applicability. In contrast, CRP, PCT and IL-6 are available in most of the hospital laboratories, and their combination in an easily computable score could improve the accuracy of sepsis diagnosis. By analyzing ROC curves in this study, we were able to select a cutoff value for the bioscore for diagnosis prediction. In each population of patients, including the total ICU population, the non-surgical subpopulation and the newly hospitalized subpopulation, this method provided a better prediction of diagnosis than any of the other biomarkers in isolation that were assessed in this study, which was similar to previous published information [13].

The present study has its limitations. First, this is a single-center study and the numbers of patients studied are limited. A larger population would add more power, making the results more generalizable. Different studies have described different ROC and AUC for the same biomarkers. This can be explained by different studied populations and laboratory techniques, all of which can impact the diagnostic and prognostic power of these tests. However, our measurements are consistent with the previous literature, validating our results. Second, we considered values only at ICU admission and we did not analyze trends during the course of stay. For example, temperature pattern can be more useful than a single value at admission [25, 26]. Moreover, other studies show how the trend and modification of biomarkers could be helpful to manage antibiotic usage [27], to monitor the patient’s recovery from sepsis and to predict the outcome. Given our aim to measure the value of a composite of clinical and laboratory data to assist the clinician toward making the correct and timely diagnosis of sepsis, in order to increase accuracy, we limited our evaluations to those at patient admission.

## Conclusions

The present study shows a combined PCT, CRP and SOFA score, used to calculate a patient bioscore, may be a valuable predictive tool to accurately diagnose sepsis.

## Additional file

10.1186/s13613-016-0153-5 The grading criteria of age, gender, SOFA, PCT, IL-6, CRP, body temperature and white blood cellcount. **Table S2.** Characteristics of infections and microorganism in septic population **Table S3.** The scoring system for sepsis diagnosis.
