# Supplementary material for: Combination of C-reactive protein, procalcitonin and sepsis-related organ failure score for the diagnosis of sepsis in critical patients
Source: Ann Intensive Care. 2016 Jun 10;6:51. doi: 10.1186/s13613-016-0153-5 (PMC4901212; doi:10.1186/s13613-016-0153-5)
Supplement: Supplementary file 1 — 10.1186/s13613-016-0153-5 The grading criteria of age, gender, SOFA, PCT, IL-6, CRP, body temperature and white blood cellcount. Table S2. Characteristics of infections and microorganism in septic population Table S3. The scoring system for sepsis diagnosis. [file 13613_2016_153_MOESM1_ESM.docx]

**Table S1** The grading criteria of age, gender, SOFA, PCT, IL-6, CRP, body temperature and white blood cell count.

| marker | grading | range |
| --- | --- | --- |
| age | 0  1 | <60  >=60 |
| gender | 0  1 | Female  Male |
| SOFA | 0  1 | <6  >6 |
| PCT (ng/ml) | 0 | <0.5 |
|  | 1 | 0.5 to 2 |
|  | 2 | 2 to 10 |
|  | 3 | >10 |
| IL-6 (U/L) | 0 | <7 |
|  | 1 | 7 to 60 |
|  | 2 | 60 to 130 |
|  | 3 | 130 to 365 |
|  | 4 | >365 |
| CRP (mg/L) | 0 | <8 |
|  | 1 | 8 to 50 |
|  | 2 | 50 to 80 |
|  | 3 | >80 |
| body temperature (°C) | 0 | 36 to 38 |
|  | 1 | <36 or >38 |
| WBC (×10^9^/L) | 0 | 4 to 12 |
|  | 1 | <4 or >12 |

**Table S2** Characteristics of infections and microorganism in septic population

|  | Septic patients (107) |
| --- | --- |
| Positive cultures - no. (%) | 67 (62.6%) |
| Multidrug resistant bacteria - no. (%) | 24 (22.4%) |
| Positive blood culture - no. (%) | 12 (11.2%) |
| *Site of infection* | |
| Lung - no. (%) | 81 (76) |
| Abdomen - no. (%) | 14 (13) |
| Blood - no. (%) | 4 (4) |
| Endocarditis - no. (%) | 5 (5) |
| Central nervous system - no. (%) | 2 (2) |
| Urinary tract - no. (%) | 1 (1) |
| Soft tissue - no. (%) | 2 (2) |
| Others - no. (%) | 8 (7) |
| Isolated pathogens - no. | 87 |
| *Bacilli (51)* | |
| [Escherichia](app:ds:escherichia)[coli](app:ds:coli)- no. (%) | 11 (13) |
| [Klebsiella pneumoniae](app:ds:Klebsiella%20pneumoniae)- no. (%) | 13 (15) |
| [Acinetobacter](app:ds:acinetobacter)[baumannii](app:ds:baumannii)- no. (%) | 12 (14) |
| [Pseudomonas aeruginosa](app:ds:pseudomonas%20aeruginosa)- no. (%) | 4 (5) |
| Others bacilli - no. (%) | 11 (13) |
| *Cocci (27)* | |
| [Coagulase negative staphylococcus](app:ds:coagulase%20negative%20staphylococcus)- no. (%) | 15 (17) |
| Staphylococcus aureus- no. (%) | 4 (5) |
| Streptococcus pneumoniae- no. (%) | 2 (2) |
| Others - no. (%) | 6 (7) |
| *Fungi (9)* | |
| Candida- no. (%) | 6 (7) |
| Others - no. (%) | 3 (3) |

**Table S3** The scoring system for sepsis diagnosis.

Step 1.

| CRP (mg/L) | score |
| --- | --- |
| <8 | 0 |
| 8-50 | 1 |
| 50-80 | 2 |
| ≥80 | 2 |

| PCT (ng/ml) | score |
| --- | --- |
| <0.5 | 0 |
| 0.5-2 | 1 |
| 2-10 | 1 |
| ≥10 | 1.5 |

| SOFA | score |
| --- | --- |
| ________ | SOFA × 0.1 |

Step 2.

| risk factor | score |
| --- | --- |
| CRP | ________ |
| PCT | ________ |
| SOFA | ________ |
|  | (+ |
| Total | ________ |
